# A Case of Status-Epilepticus-Associated Transient Hyperammonemia in the Emergency Department

**DOI:** 10.1155/2017/9436095

**Published:** 2017-12-24

**Authors:** Brittany Pelsue, Jonathan G. Rogg

**Affiliations:** Department of Pharmacy, Memorial Hermann-Texas Medical Center, 6411 Fannin Street, Houston, TX 77030, USA

## Abstract

This report describes a case of transient hyperammonemia following tonic-clonic status epilepticus with an initial ammonia level of 537 Umol/L. This appears to be the highest transient ammonia level reported in the literature in this clinical scenario. This is an affirmation that an initial elevated ammonia level in the absence of hepatic dysfunction should be interpreted with caution when associated with status epilepticus. Repeat levels should be drawn to identify transient hyperammonemia and determine the need for treatment if levels do not decrease.

## 1. Introduction

In patients presenting to the emergency department with seizure-like activity, ammonia levels may be part of the initial workup. Ammonia is a precursor of glutamine, which is a substrate required for normal mitochondrial functioning. Ammonia also plays a role in the metabolism and recycling of neurotransmitters, as well as in acid base balance since sodium bicarbonate is created with its excretion. Ammonia is normally present in the blood at low levels, typically less than 40 Umol/L. When ammonia accumulates it is a neurotoxin, causing astrocyte edema and inflammation [1–3]. Hyperammonemia can result from excess production or impaired elimination of ammonia, which can be seen in acute or chronic hepatic failure, multiple myeloma, urea cycle defects, fatty acid oxidation defects, Reye's syndrome, or exposure to toxins or medications such as valproic acid of 5-fluorouracil [1].

Hyperammonemia is a widely recognized cause of seizures due to the neurotoxic effects. When it is identified as a cause of seizures, elevated ammonia levels must be lowered, often using pharmacologic measures. It has been theorized that seizures themselves can paradoxically elevate ammonia levels. This occurs most commonly in the setting of generalized tonic-clonic seizures, where muscle contractions lead to adenosine monophosphate deamination and the subsequent production of ammonia [4]. Once muscle contractions cease, if liver function is maintained, the ammonia will be cleared and levels will return to normal. In a prospective observational study of 121 patients it was described that, in transient hyperammonemia, ammonia levels return to normal within 8 hours [4]. In this same study, it was found that transient hyperammonemia was associated with tonic-clonic seizures, male gender, diabetes, and alcohol related seizures. The range of ammonia detection in this study was 94 mcg/dL (67 Umol/L)–250 mcg/dL (178 Umol/L), with previous case reports documenting levels up to 392 mcg/dL (279.8 Umol/L) [5]. It has been established in the adult population that, because of the temporary nature of this laboratory abnormality, transient hyperammonemia in this setting does not require intervention.

## 2. Case Presentation

A 29-year-old woman presented to the emergency department at a large, academic medical center with an acute onset of tonic-clonic seizures. She has a past medical history of bipolar disorder for which she takes lithium, and had used cocaine and methamphetamine prior to arrival. She received a total of 4 mg of lorazepam intravenous (IV), which did not abate the seizure. She was given a loading dose of levetiracetam after which clinical signs of seizure activity ceased, resulting in a seizure time of about 35 minutes (15 minutes in-hospital, with reported onset 20 minutes prior to arrival). She then developed supraventricular tachycardia (SVT) with a heart rate greater than 170 beats per minute which converted to normal sinus rhythm following a total of 18 mg IV adenosine.

The ammonia level taken on admission was 537 Umol/L using the Siemens Dimension Vista® system (upper limit of normal [ULN] ≤ 45 Umol/L). Her liver function tests were within normal limits with the exception of a mildly elevated aspartate transaminase (AST) of 62 units/L (ULN 37 units/L) which could be explained by muscle breakdown during the seizure. The urine drug screen was positive for amphetamines and her lithium level was subtherapeutic at 0.31 mEq/L. She suffered from multiple laboratory derangements including hypernatremia (150 mEq/L), as well as other findings consistent with seizures such as an anion gap metabolic acidosis. Labs were repeated once the seizures abated and the ammonia had decreased to 351 Umol/L, just 30 minutes after the previous level. She had not received IV fluids or ammonia-lowering therapy during this time. Almost 6 hours after the initial ammonia was drawn, still having received no ammonia-lowering therapy, the ammonia level was 68 Umol/L. The patient did not experience further seizure-like activity while in the emergency department and was admitted for workup of metabolic derangements. She was discharged five days later without sequelae.

## 3. Discussion

This report describes a case of transient hyperammonemia in the setting of acute-onset status epilepticus. This is a subset of patients separate from those experiencing a gradual onset of encephalopathy over two to three days, in whom prompt treatment of initial hyperammonemia may be appropriate. The patient ingested cocaine and methamphetamines prior to arrival, both of which have stimulant effects and have the potential to have caused her seizure activity. Neither of these illicit drugs has a causal link to hyperammonemia, although a case report described an ammonia level of 91 Umol/L following methamphetamine use [6]. This is likely not the cause of this lab abnormality in this patient due to the long half-life of methamphetamine in the setting of a rapidly declining ammonia level. She was taking no other medications that are associated with elevated ammonia levels.

Aside from the tonic-clonic nature of her seizures, this patient does not fit other previously described risk factors associated with seizure-related hyperammonemia including male gender, diabetes, and alcohol related seizures. Previous case reports have noted initial ammonia levels up to 280 Umol/L but have not documented ammonia greater than 500 Umol/L in this setting. This adds to the body of evidence by allowing an alternative explanation for this extreme laboratory abnormality and drawing attention to the possibility that transient hyperammonemia can affect those outside of previously recognized demographics. Transient hyperammonemia following the acute onset of status epilepticus does not require treatment and if recognized can help to avoid unnecessary interventions.

## 4. Conclusion

This report documents an initial ammonia level higher than those previously recognized in the setting of transient hyperammonemia following status epilepticus. This shows that even severely elevated values may result from, rather than cause, seizures. Levels should return to near-normal within 8 hours of initial presentation, or alternative causes should be considered.
